# Brain transcriptome analysis of a familial Alzheimer’s disease-like mutation in the zebrafish *presenilin 1* gene implies effects on energy production

**DOI:** 10.1186/s13041-019-0467-y

**Published:** 2019-05-03

**Authors:** Morgan Newman, Nhi Hin, Stephen Pederson, Michael Lardelli

**Affiliations:** Department of Molecular and Biomedical Science, University of Adelaide, School of Biological Sciences, North Terrace, Adelaide, SA 5005 Australia

**Keywords:** Alzheimer’s disease, Presenilin 1, Mutation, Transcriptome, Brain, ATP synthesis, Mitochondria, Vacuolar acidification, Zebrafish, Genome editing

## Abstract

To prevent or ameliorate Alzheimer’s disease (AD) we must understand its molecular basis. AD develops over decades but detailed molecular analysis of AD brains is limited to postmortem tissue where the stresses initiating the disease may be obscured by compensatory responses and neurodegenerative processes. Rare, dominant mutations in a small number of genes, but particularly the gene *PRESENILIN 1* (*PSEN1*), drive early onset of familial AD (EOfAD). Numerous transgenic models of AD have been constructed in mouse and other organisms, but transcriptomic analysis of these models has raised serious doubts regarding their representation of the disease state. Since we lack clarity regarding the molecular mechanism(s) underlying AD, we posit that the most valid approach is to model the human EOfAD genetic state as closely as possible. Therefore, we sought to analyse brains from zebrafish heterozygous for a single, EOfAD-like mutation in their *PSEN1*-orthologous gene, *psen1*. We previously introduced an EOfAD-like mutation (Q96_K97del) into the endogenous *psen1* gene of zebrafish. Here, we analysed transcriptomes of young adult (6-month-old) entire brains from a family of heterozygous mutant and wild type sibling fish. Gene ontology (GO) analysis implies effects on mitochondria, particularly ATP synthesis, and on ATP-dependent processes including vacuolar acidification.

**Electronic supplementary material:**

The online version of this article (10.1186/s13041-019-0467-y) contains supplementary material, which is available to authorized users.

## Background

AD is the most common form of dementia with severe personal, social, and economic impacts. Rare, familial forms of AD exist caused by autosomal dominant mutations in single genes (reviewed by [1]). The majority of these mutations occur in the gene *PRESENILIN 1* (*PSEN1*) that encodes a multipass integral membrane protein involved in intra-membrane cleavage of numerous proteins [1].

A wide variety of transgenic models of AD have been created and studied. These are aimed at reproducing histopathologies posited to be central to the disease process, i.e. amyloid plaques and neurofibrillary tangles of the protein MAPT [2]. However, analysis of the effects on the brain transcriptome of the transgenes driving a number of these mouse models showed little concordance with transcriptomic differences between human AD brains and age-matched controls [3] (although a recent study asserts that this lack of concordance for the popular “5XFAD” transgenic mouse model is due to previous failure to analyse the effects of its transgenes in a variety of genetic backgrounds [4]). We posit that, in the absence of an understanding of the molecular mechanism(s) underlying AD, the most objective approach to modeling this disease (or, at least, modeling its genetic form, EOfAD) is to create a genetic state as similar as possible to the EOfAD state in humans. Mouse “knock-in” models of EOfAD mutations were created over a decade ago and showed subtle phenotypic effects but not the desired histopathologies (e.g. [5, 6]). However, at that time, researchers did not have access to RNA-Seq technology. To the best of our knowledge, transcriptome analysis of the EOfAD mutation knock-in mouse models was never performed.

In humans, AD is thought to develop over decades and the median survival to onset age for EOfAD mutations in human *PSEN1* considered collectively is 45 years [7]. Functional MRI of human children carrying EOfAD mutations in *PSEN1* has revealed differences in brain activity compared to non-carriers in individuals as young as 9 years of age [8]. Presumably therefore, heterozygosity for EOfAD mutations in *PSEN1* causes early molecular changes/stresses that eventually lead to AD.

Transcriptome analysis is currently the most detailed molecular phenotypic analysis possible on cells or tissues. Here we present an initial analysis of the transcriptomic differences caused in young adult (6-month-old) zebrafish brains by the presence of an EOfAD-like mutation in the gene *psen1* that is orthologous to the human *PSEN1* gene. GO analysis supports very significant effects on mitochondrial function, especially synthesis of ATP, and on ATP-dependent functions such as the acidification of lysosomes that are critical for autophagy.

## Materials and methods

The mutant allele, Q96_K97del, of *psen1* was a byproduct identified during our introduction of the K97fs mutation into *psen1* (that models the K115fs mutation of human *PSEN2* – see [9] for an explanation).

Q96_K97del is a deletion of 6 nucleotides from the coding sequence of the *psen1* gene. This is predicted to distort the first lumenal loop of the Psen1 protein. In this sense, it is similar to a number of EOfAD mutations of human *PSEN1* [10]. Also, in common with all the widely distributed EOfAD mutations in *PSEN1*, (and consistent with the PRESENILIN EOfAD mutation “reading frame preservation rule” [1]), the Q96_K97del allele is predicted to encode a transcript that includes the C-terminal sequences of the wild type protein. Therefore, as a model of an EOfAD mutation, it is superior to the K97fs mutation in *psen1* [9].

To generate a family of heterozygous Q96_K97del allele (i.e. *psen1*^*Q96_K97del*^/+) and wild type (+/+) sibling fish, we mated a *psen1*^*Q96_K97del*^/+ individual with a +/+ individual and raised the progeny from a single spawning event together in one tank. Zebrafish can live for up to 5 years but, in our laboratory, typically show greatly reduced fertility after 18 months. The fish become fertile after around 3 months of age, so we regard 6-month-old fish as equivalent to young adult humans. Therefore we analysed the transcriptomes of entire young adult, 6-month-old fish brains using poly-A enriched RNA-seq technology, and estimated gene expression from the resulting single-end 75 bp reads using the reference GRCz11 zebrafish assembly transcriptome [11, 12]. Each zebrafish brain has a mass of approximately 7 mg. Since AD is more prevalent in human females than males, and to further reduce gene expression “noise” in our analyses, we obtained brain transcriptome data from four female wild type fish and four female heterozygous mutant fish. This data has been made publicly available at the Gene Expression Omnibus (GEO, see under *Availability of data and materials* below).

## Results

### Differentially expressed genes (DE genes)

Genes differentially expressed between wild type and heterozygous mutant sibling fish were identified using moderated *t*-tests and a false discovery rate (FDR)-adjusted *p*-value cutoff of 0.05 as previously described [9, 13, 14]. In total, 251 genes were identified as differentially expressed (see Additional file 1). Of these, 105 genes showed increased expression in heterozygous mutant brains relative to wild type sibling brains while 146 genes showed decreased expression.

### GO analysis

To understand the significance for brain cellular function of the differential gene expression identified in young adult heterozygous mutant brains we used the *goana* function [15] of the *limma* package of Bioconductor software [14] to identify GOs in which the DE genes were enriched at an FDR-corrected *p*-value of less than 0.05. Seventy-eight GOs were identified (Table 1) of which 20 addressed cellular components (CC). Remarkably, most of these CCs concerned the mitochondrion, membranes, or ATPases. Seventeen GOs addressed molecular functions (MF) and largely involved membrane transporter activity, particularly ion transport and ATPase activity coupled to such transport. Forty-one GOs addressed biological processes (BP) and involved ATP metabolism, ribonucleoside metabolism, and transmembrane transport processes including vacuolar acidification (that has previously been identified as affected by EOfAD mutations in *PSEN1* [16]). Overall, our GO analysis indicates that this EOfAD-like mutation of zebrafish *psen1* has very significant impacts on cellular energy metabolism and transmembrane transport processes.

**Table 1:** GOs enriched for genes differentially expressed between heterozygous mutant and wild type sibling fish brains

Gene Ontology Term	Ontology	Total Genes	DE Genes	p-value	FDR *p*-value
ATP biosynthetic process	BP	29	7	3.48987E-08	0.00041
ribonucleoside triphosphate biosynthetic process	BP	49	8	9.41317E-08	0.00045
nucleoside triphosphate biosynthetic process	BP	54	8	2.06555E-07	0.00060
purine nucleoside triphosphate biosynthetic process	BP	41	7	4.46237E-07	0.00060
purine ribonucleoside triphosphate biosynthetic process	BP	41	7	4.46237E-07	0.00060
hydrogen transport	BP	60	8	4.783E-07	0.00060
proton transport	BP	60	8	4.783E-07	0.00060
energy coupled proton transport, down electrochemical gradient	BP	27	6	5.89038E-07	0.00060
ATP synthesis coupled proton transport	BP	27	6	5.89038E-07	0.00060
transport	BP	2072	48	2.11748E-06	0.00165
purine nucleoside monophosphate biosynthetic process	BP	54	7	3.09019E-06	0.00172
purine ribonucleoside monophosphate biosynthetic process	BP	54	7	3.09019E-06	0.00172
hydrogen ion transmembrane transport	BP	54	7	3.09019E-06	0.00172
ribonucleoside triphosphate metabolic process	BP	133	10	3.8448E-06	0.00178
establishment of localization	BP	2123	48	4.20295E-06	0.00182
ATP metabolic process	BP	109	9	5.50772E-06	0.00230
nucleoside triphosphate metabolic process	BP	140	10	6.08925E-06	0.00245
cation transport	BP	452	18	6.61154E-06	0.00258
monovalent inorganic cation transport	BP	219	12	1.10729E-05	0.00392
ribonucleoside monophosphate biosynthetic process	BP	65	7	1.08944E-05	0.00392
nucleoside monophosphate biosynthetic process	BP	68	7	1.47269E-05	0.00492
purine ribonucleoside triphosphate metabolic process	BP	125	9	1.68142E-05	0.00546
purine nucleoside triphosphate metabolic process	BP	126	9	1.79263E-05	0.00552
transmembrane transport	BP	654	21	2.93288E-05	0.00797
purine nucleoside monophosphate metabolic process	BP	136	9	3.2951E-05	0.00837
purine ribonucleoside monophosphate metabolic process	BP	136	9	3.2951E-05	0.00837
energy coupled proton transmembrane transport, against electrochemical gradient	BP	35	5	5.20342E-05	0.01106
ATP hydrolysis coupled proton transport	BP	35	5	5.20342E-05	0.01106
ATP hydrolysis coupled transmembrane transport	BP	35	5	5.20342E-05	0.01106
ATP hydrolysis coupled ion transmembrane transport	BP	35	5	5.20342E-05	0.01106
ATP hydrolysis coupled cation transmembrane transport	BP	35	5	5.20342E-05	0.01106
ion transport	BP	737	22	5.61478E-05	0.01152
localization	BP	2621	52	6.0913E-05	0.01207
ribonucleoside monophosphate metabolic process	BP	147	9	6.06496E-05	0.01207
nucleoside monophosphate metabolic process	BP	150	9	7.09445E-05	0.01360
single-organism localization	BP	819	23	9.51294E-05	0.01738
single-organism transport	BP	776	22	0.000119082	0.02109
ribonucleotide biosynthetic process	BP	129	8	0.000143028	0.02423
ribose phosphate biosynthetic process	BP	129	8	0.000143028	0.02423
vacuolar acidification	BP	11	3	0.000246582	0.04101
ribonucleotide metabolic process	BP	220	10	0.000281352	0.04506
proton-transporting two-sector ATPase complex, proton-transporting domain	CC	25	6	3.59375E-07	0.00060
proton-transporting two-sector ATPase complex	CC	45	7	8.65692E-07	0.00078
mitochondrial membrane	CC	285	15	1.42199E-06	0.00119
mitochondrial envelope	CC	303	15	3.0322E-06	0.00172
membrane part	CC	4868	85	1.1722E-05	0.00403
organelle membrane	CC	789	24	1.84982E-05	0.00555
mitochondrial inner membrane	CC	195	11	1.97958E-05	0.00579
integral component of membrane	CC	4419	78	2.52479E-05	0.00720
intrinsic component of membrane	CC	4453	78	3.37749E-05	0.00840
organelle envelope	CC	420	16	3.76291E-05	0.00917
envelope	CC	422	16	3.98337E-05	0.00950
organelle inner membrane	CC	215	11	4.86028E-05	0.01106
Cul2-RING ubiquitin ligase complex	CC	7	3	5.4156E-05	0.01131
proton-transporting ATP synthase complex	CC	19	4	6.25883E-05	0.01220
mitochondrial membrane part	CC	117	8	7.21148E-05	0.01360
mitochondrial part	CC	404	15	8.83156E-05	0.01639
membrane	CC	5379	88	0.000106964	0.01924
vacuolar proton-transporting V-type ATPase, V0 domain	CC	9	3	0.000127733	0.02229
mitochondrial proton-transporting ATP synthase complex, coupling factor F(o)	CC	12	3	0.000325933	0.04885
proton-transporting V-type ATPase, V0 domain	CC	12	3	0.000325933	0.04885
ATPase activity, coupled to transmembrane movement of ions, rotational mechanism	MF	34	7	1.1446E-07	0.00045
hydrogen ion transmembrane transporter activity	MF	84	9	6.11883E-07	0.00060
ATPase activity, coupled to transmembrane movement of substances	MF	98	9	2.27123E-06	0.00166
hydrolase activity, acting on acid anhydrides, catalyzing transmembrane movement of substances	MF	101	9	2.92425E-06	0.00172
primary active transmembrane transporter activity	MF	104	9	3.73269E-06	0.00178
P-P-bond-hydrolysis-driven transmembrane transporter activity	MF	104	9	3.73269E-06	0.00178
cation-transporting ATPase activity	MF	56	7	3.96731E-06	0.00178
ATPase coupled ion transmembrane transporter activity	MF	56	7	3.96731E-06	0.00178
ATPase activity, coupled to movement of substances	MF	112	9	6.88692E-06	0.00260
active ion transmembrane transporter activity	MF	96	8	1.72916E-05	0.00546
active transmembrane transporter activity	MF	281	13	2.87859E-05	0.00797
proton-transporting ATP synthase activity, rotational mechanism	MF	16	4	3.02121E-05	0.00803
transporter activity	MF	991	25	0.000249051	0.04101
substrate-specific transmembrane transporter activity	MF	709	20	0.000263528	0.04279
ion transmembrane transporter activity	MF	660	19	0.000293184	0.04572
substrate-specific transporter activity	MF	828	22	0.000297009	0.04572
monovalent inorganic cation transmembrane transporter activity	MF	264	11	0.000297217	0.04572

GOs are grouped by ontology (BP, CC or MF) and ranked by FDR-corrected *p*-value

## Additional file

Additional file 1: Genes differentially expressed between heterozygous mutant and wild type brains at 6 months. Lists the genes identified as differentially expressed between the brains of heterozygous *psen1*^*Q96_K97del*^ mutant fish and the brains of their wild type siblings at an age of 6 months. Genes are ranked according to FDR-corrected *p*-value. Only genes with a FDR-corrected p-value less than 0.05 are shown. “FC” denotes fold change. “DE” denotes differential expression. For DE_Direction, “1” denotes increased expression in the mutant and “-1” denotes decreased expression in the mutant. (XLSX 39 kb)

## Abbreviations

AD: Alzheimer’s disease
ATP: Adenosine triphosphate
BP: Biological process (GO term)
CC: Cellular component (GO term)
DE genes: Differentially expressed genes
EOfAD: Early onset familial Alzheimer’s disease
FDR: False discovery rate
GEO: Gene Expression Omnibus
GO: Gene ontology
MAPT: MICROTUBULE-ASSOCIATED PROTEIN TAU (human protein)
MF: Molecular function (GO term)
mg: Milligrams
MRI: Magnetic resonance imaging
*PSEN1*: *PRESENILIN 1* (human gene)
PSEN1: PRESENILIN 1 (human protein)
*psen1*: *presenilin 1* (zebrafish gene)
Psen1: Presenilin 1 (zebrafish protein)
